# Editorial: From bench to bedside: the challenge of chronic pain

**DOI:** 10.3389/fpain.2023.1271468

**Published:** 2023-08-17

**Authors:** Maria Maiarú, Amelia Hollywood, Michele Trimboli

**Affiliations:** ^1^ School of Pharmacy, University of Reading, Reading, United Kingdom; ^2^ Institute of Neurology, AOU Renato Dulbecco, Catanzaro, Italy

**Keywords:** pain, chronic pain, translational research, pain treatment, from bench to bed, patient experience

**Editorial on the Research Topic** From bench to bedside: the challenge of chronic pain

Chronic pain affects millions of people worldwide and presents a huge social and economic burden (1). Opioids and non-opioid drugs have become popular over the past few decades; however, the effective pain relief they provide is only partial, and their use is frequently associated with severe side effects (2).

Pain has two main components, namely, a sensory and an affective or “emotional” component (3), and both should be considered when searching for new analgesics. While there is variability in the patient experience of chronic pain and the available treatments, understanding the lived experience is key to ensuring that new treatments improve quality of life. The articles included in this Research Topic provide new insights into a wide range of research on chronic pain from pain mechanism to the experience of the patient living with pain, thus highlighting translation from bench to bedside.

## New treatments

Chemotherapy-induced peripheral neuropathy (CIPN) is the major dose-limiting side effect of most anticancer drugs, with pain being the most reported symptom (4). The work of Takeshita and colleagues demonstrated the efficacy of soluble epoxide hydrolase inhibitor (sEHI) oral administration in improving mechanical hypersensitivity in different models of CIPN induced by commonly used anticancer drugs (Takeshita et al., 2023). Mitochondrial dysfunction has been suggested as a cellular mechanism underlying CIPN (5). sEHI can reduce mitochondrial stress, and safety has been demonstrated in a Phase 1 clinical trial in human volunteers (6). sEHI can also reduce inflammation and pain in dogs with osteoarthritis (7) and attenuate chronic pain in a murine model of diabetic neuropathy (8). This research could open the way to the development of effective CIPN treatments.

Acupuncture analgesia, a time-honoured treatment, is a captivating paradox—believed to provide relief but without strong scientific evidence. Its purported link to the central nervous system, encompassing diverse brain regions from the cortex to the brain stem, has long piqued the interest of researchers. However, the question lingers: do these regions act in unison or as multiple sub-units with distinct functions? In a bid to decipher this enigma, the advent of magnetoencephalography (MEG) holds promise. This cutting-edge neuroimaging technique offers unprecedented insights into the oscillatory frequency of neural signals and brain regions (9). Here, Kato and colleagues identified two brain regions involved in acupuncture-mediated analgesia (Kato et al., 2022). They investigated the resting-state brain activity of 21 chronic pain patients before and after acupuncture treatment. This comprehensive approach encompassed subjective pain assessment through a visual analogue scale and precise identification of brain regions and frequencies associated with acupuncture analgesia. The results revealed two distinct groups of resting-state brain activity changes, namely, low-frequency oscillatory activity in specific regions and high-frequency oscillatory activity in others. This intriguing revelation suggests that acupuncture analgesia may influence two or more sub-units of the neural systems, providing insight into the underlying neural mechanisms.

## Patient experience

Gaining insight into the pain mechanisms and treatment provides patients and clinicians with the most advanced understanding of this complex condition to ensure optimal outcomes. It is also essential to consider the patient's experience living with and managing their condition.

The negative emotional and sensory experience associated with chronic pain is complex, and a lifetime of experiences can influence clinical outcomes. Tidmarsh and colleagues highlighted the importance of childhood experiences on outcomes for patients with chronic pain in adulthood (Tidmarsh et al., 2022). The review identified the literature that explores the impact of adverse childhood experiences (ACEs) in pain management. ACEs are potentially traumatic encounters before 18 years of age (10). The review highlighted that ACEs are associated with an increased risk of chronic pain and poorer clinical outcomes. Therefore, when clinicians assist patients with chronic pain, they must ensure that they provide patient-centred care and identify any relevant past experiences, along with current symptoms, to provide the best care and achieve optimal outcomes.

Chronic pain can be a standalone clinical problem or coexist with an underlying medical condition. The variability in outcomes highlights the need to explore a variety of presentations in a wide range of populations. In their study, Alkandari and Hollywood explored the experiences of people living with peripheral neuropathy (Alkandari and Hollywood, 2023). This study provided novel insights into the lived experience of patients in a non-Western country, which provides a global perspective on a condition that affects millions of people worldwide. The experiences of people living with peripheral neuropathy in Kuwait were investigated, further highlighting the importance of a patient-centred approach. The authors suggest that adequate support is needed to promote self-efficacy, i.e., confidence in one's ability to perform a particular behaviour (11) and encourage self-management strategies. Long-term, chronic conditions require a holistic approach to support patients and improve their quality of life.

Understanding patients’ experiences, to enable patient-centred care, is key to supporting pain management. Pain management programmes (PMPs) are offered worldwide to help patients manage their chronic pain and improve their quality of life (12). The patient experience of PMPs must be better studied to improve this treatment approach and improve outcomes. Finlay and colleagues explored patient-to-patient interactions during a 6-week PMP (Finlay et al., 2022). This programme included two 4-h face-to-face sessions per week for adults living with chronic pain, and each session content was based on the recommended guidelines (13). The patient-to-patient interactions highlighted the importance of venting and humour relating to chronic pain, in building social support and making sense of their pain experiences. This offers new insights into the mechanisms of action of PMPs beyond the content delivered to the interactions between patients, which can also influence clinical outcomes.

## Conclusion

There is an urgent need for better and safer chronic pain treatments. Promising preclinical research is published every year; however, positive animal data generally have not translated well to pain relief in humans. More translational research is required to bridge the gap between “bench” and “bedside,” which is an essential step towards a new era of pain treatment.
